# Sexual complementarity between host humoral toxicity and soldier caste in a polyembryonic wasp

**DOI:** 10.1038/srep29336

**Published:** 2016-07-07

**Authors:** Daisuke Uka, Takuma Sakamoto, Jin Yoshimura, Kikuo Iwabuchi

**Affiliations:** 1 Faculty of Agriculture, Tokyo University of Agriculture and Technology, Fuchu, Tokyo 183-8509, Japan; 2 Graduate School of Science and Technology and Department of Mathematical and Systems Engineering, Shizuoka University, Hamamatsu, 432-8561, Japan; 3 Marine Biosystems Research Center, Chiba University, Kamogawa, Chiba 299-5502, Japan; 4 Department of Environmental and Forest Biology, State University of New York College of Environmental Science and Forestry, Syracuse, NY 13210, USA

## Abstract

Defense against enemies is a type of natural selection considered fundamentally equivalent between the sexes. In reality, however, whether males and females differ in defense strategy is unknown. Multiparasitism necessarily leads to the problem of defense for a parasite (parasitoid). The polyembryonic parasitic wasp *Copidosoma floridanum* is famous for its larval soldiers’ ability to kill other parasites. This wasp also exhibits sexual differences not only with regard to the competitive ability of the soldier caste but also with regard to host immune enhancement. Female soldiers are more aggressive than male soldiers, and their numbers increase upon invasion of the host by other parasites. In this report, *in vivo* and *in vitro* competition assays were used to test whether females have a toxic humoral factor; if so, then its strength was compared with that of males. We found that females have a toxic factor that is much weaker than that of males. Our results imply sexual complementarity between host humoral toxicity and larval soldiers. We discuss how this sexual complementarity guarantees adaptive advantages for both males and females despite the one-sided killing of male reproductives by larval female soldiers in a mixed-sex brood.

Sexual selection often results in sex differences regarding certain traits (i.e., secondary sexual traits)^1^. However, sex differences are not expected in the traits determined by natural selection because this process usually operates on males and females evenly. Here, we consider the sex differences concerning defense against an enemy (a naturally selected trait). In a host multi-parasitized by different parasitic wasp species (usually called parasitoids), interspecific conflict arises for host resources. In general, intrinsic interspecific competition between parasitic wasps is performed via physical attacks, physiological suppression, or both^2^,^3^,^4^. For example, the larvae of certain solitary parasitoids have large fighting mandibles to eliminate heterospecific competitors. The larvae of most gregarious and solitary parasitoids also eliminate competitors using physiological suppression via toxic factors, anoxia induction or nutritional removal.

Most polyembryonic parasitoids with a prolonged embryonic stage lack an aggressive larval form that include features such as a large mandible during its development in a host. However, the polyembryonic parasitoids of the genus *Copidosoma* and other closely related encyrtid genera have sterile larval soldiers that provide a unique defense against heterospecific and conspecific competitors. Of these soldiers, *Copidosoma floridanum* is a well-studied polyembryonic parasitoid of Plusiine moths (e.g., *Acanthoplusia agnata*). A female parasitic wasp lays one or two eggs into a host egg. The host egg hatches and undergoes six larval instars, during which, the parasitoid embryos proliferate clonally to produce more than 2,000 embryos. Most of them develop into reproductive larvae within the sixth (final) instar host larva, and they eventually emerge as adult wasps. Brood size (the number of adults emerging from the same host) is generally positively correlated with the maximum weight of the host larva which represents the host carrying capacity^5^,^6^. In a mixed-sex brood, which is derived from female and male eggs laid in the same host egg, the sex ratio is often extremely female-biased because most male embryos are killed by female soldier larvae. Note that those male embryos were to grow to male reproductive if not killed. In the mixed-sex brood, adults mate before disperse from the dead host, from which they emerge^7^. However, a smaller number of embryos develop precociously into larval soldiers that are morphologically and behaviorally distinct. These soldiers do not molt and eventually die without pupating^6^,^7^,^8^,^9^.

As in most hymenopteran species, unfertilized (haploid) eggs produce males, and fertilized (diploid) eggs produce females. Soldier larvae are formed in both sexes, but the aggressiveness in male soldiers are much weaker than female soldiers in the Japanese strain^10^ and completely lost in the American strain^11^. In the mixed sex broods of *C. floridanum*, sexual conflict occurs between the sexes of non-relatives to create a female-biased sex ratio. Sex differences in the level of aggressiveness between female and male soldier larvae likely evolved because of sexual conflict^7^,^11^,^12^,^13^. The sex difference in aggressiveness in soldier larvae should increase the protective ability of both sexes against heterospecific competitors^10^.

Interestingly, however, when the hosts are multiparasitized by *C. floridanum* and *Glyptapanteles pallipes*, *C. floridanum* adults are usually produced without differences in numbers of males and females. Thus, no difference is present in the final successful parasitism between the sexes despite the notable difference in the aggressiveness of the soldier larvae, suggesting the existence of an additional factor regarding the elimination of heterospecific competitors^6^,^10^. Our previous study of the male broods of *C. floridanum* derived from unfertilized eggs showed that the parasitized host hemolymph contained a humoral factor that was toxic to *G. pallipes* larvae. The toxicity was also found in the hosts without soldier larvae^14^. That is, the male toxic humoral factor may be independent to the soldier larvae and is likely an addition to the soldier larvae in the intrinsic interspecific competition of *C. floridanum*. To date, a sex difference exists in the aggressiveness of soldier larvae^10^; however, whether sex differences exist in the humoral factor is unknown because the toxicity of the female humoral factor has not been reported.

Theory predicts that females invest more in longevity than males^15^,^16^. Given that both soldiers and humoral factors have evolved in the same context and that a sex difference is observed in soldier larvae, sex differences should also be expected with regard to the toxicity of the humoral factor.

This study examined the toxicity of host hemolymph parasitized by female *C. floridanum* against the eggs and larvae of the braconid parasitoid, G. *pallipes*. We compare the results of this study with our previous reports regarding the male humoral factor. We then discuss the defense system of the parasitoid wasp as an adaptive system. We also provide a new adaptive interpretation for the killing of male embryos by female soldier larvae.

## Results

**Figure 1:**
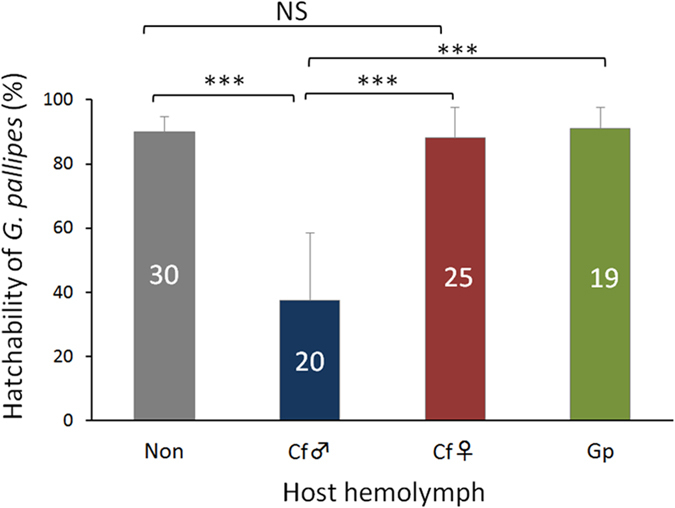
Hatchability of *G. pallipes* in the host injected with experimental host hemolymph. L3D1 host larvae were parasitized by *G. pallipes* and subsequently injected with 6 μl of host hemolymph that was either non-parasitized (Non) or parasitized by male (Cf♂) or female (Cf♀) *C. floridanum* or *G. pallipes* (Gp) just after parasitism. Hatchability was investigated after 48 h of parasitism. The numbers of hosts per treatment are shown in each bar. The data are presented as the mean ± SD. Statistical analyses were performed using the Welch t-test (***p < 0.001; NS, not significant).

The toxicity of the hemolymph taken from a host parasitized by male or female *C. floridanum* to *G. pallipes* eggs was examined via hemolymph injection into the host larvae immediately after parasitism by *G. pallipes*. Male *C. floridanum*–parasitized host hemolymph killed approximately 75% of the *G. pallipes* eggs. In contrast, female *C. floridanum*-parasitized host hemolymph invoked no apparent ovicidal effect, similar to the hemolymph from nonparasitized hosts (control 1) or *G. pallipes*-parasitized hosts (control 2; Fig. 1, Supplementary Fig. 1, Supplementary Table 1–2).

**Figure 2:**
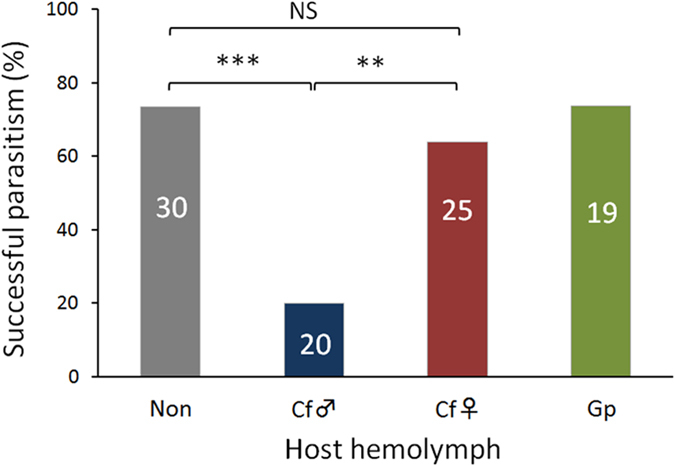
Successful parasitism of G. pallipes in the host injected with experimental host hemolymph. The successful parasitism in the host injected with 6 μl of host hemolymph that was either non-parasitized (Non) or parasitized by male (Cf♂) or female (Cf♀) *C. floridanum* or *G. pallipes* (Gp) was evaluated by the rate at which *G. pallipes* larvae egressed to pupate from a host. The numbers of hosts per treatment are shown in each bar. Statistical analyses were performed using the Welch t-test (***p < 0.001, **p < 0.01; NS, not significant).

We evaluated the successful parasitism of the host by *G. pallipes* based on the rate at which *G. pallipes* larvae egressed to pupate. The ovicidal effect of the male *C. floridanum*–parasitized host hemolymph significantly affected the successful parasitism of *G. pallipes*, whereas the female *C. floridanum*–parasitized host hemolymph exhibited no significant effect (Fig. 2, Supplementary Table 3). These results indicate that female *C. floridanum*-parasitized host hemolymph has no apparent ovicidal effect on *G. pallipes* eggs.

**Figure 3:**
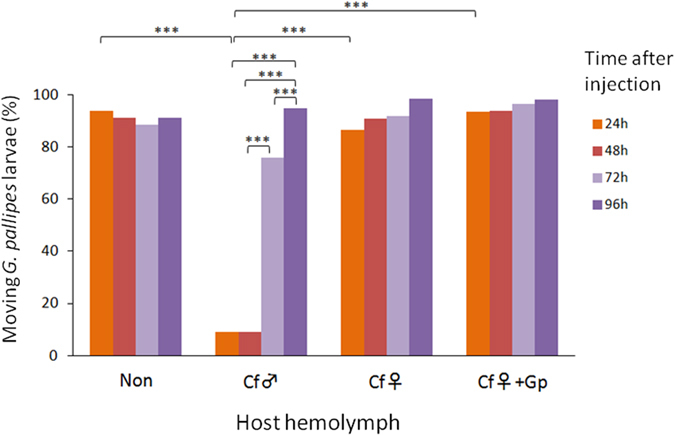
The effect of host hemolymph injection on *G. pallipes* larval movement. L3D1 host larvae were parasitized by *G. pallipes* and subsequently injected with 6 μl of host hemolymph that was either non-parasitized (Non) or parasitized by male (Cf♂) or female (Cf♀) *C. floridanum* or *G. pallipes* (Gp) after 72 h of parasitism. The data for hemolymph from nonparasitized hosts and male *C. floridanum*-parasitized hosts were partly reported^14^. Statistical analyses were performed using the Welch t-test (***p < 0.001).

We also evaluated the paralysis of the *G. pallipes* larvae via the *C. floridanum*–parasitized host hemolymph using an *in vivo* assay and observing the rate of movement in the *G. pallipes* larvae at 24, 48, 72 and 96 hours after the injection of the experimental host hemolymph. Male *C. floridanum*–parasitized host hemolymph invoked paralysis in *G. pallipes* larvae at 24 and 48 hours after injection; however, the larvae exhibited recovery from paralysis at 72 and 96 hours. In contrast, female *C. floridanum*-parasitized host hemolymph had no apparent effect at any point, similar to the finding observed in the two controls (i.e., the hemolymph from the non-parasitized hosts and from [female *C. floridanum*^+^ *G. pallipes*]-parasitized hosts; Fig. 3, Supplementary Tables 4 and 5). These results indicate that female *C. floridanum*-parasitized host hemolymph has no apparent paralysis effect on *G. pallipes* larvae.

**Figure 4:**
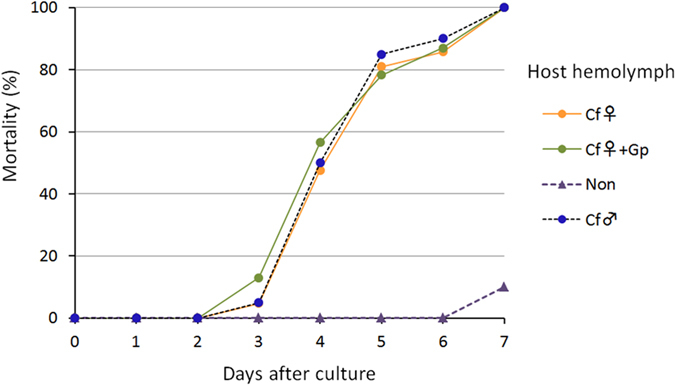
*In vitro* effects of the experimental host hemolymph on *G. pallipes* larval mortality. Seven-day-old *G. pallipes* larvae were individually cultured in 20-μl drops of MGM450 medium. The hemolymph collected from non-parasitized (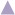), female *C. floridanum*-parasitized (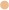) or multiparasitized hosts (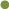) was added to the medium to achieve a final concentration of 10%. The data for the *in vitro* culture with hemolymph from the non-parasitized host (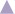) and male *C. floridanum*-parasitized hosts (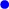, dashed line) were previously reported^14^. The larvae were cultured at 25 °C for 7 days. The *G. pallipes* larvae stages (survival or death) were observed and recorded daily.

In a previous report^14^, we showed that the hemolymph from the hosts parasitized by male *C. floridanum* had *in vitro* toxicity, and all *G. pallipes* larvae died within 7 days of culture. To examine the sex difference in the toxicity of parasitized host hemolymph, we assayed the toxicity of the host hemolymph parasitized by female *C. floridanum*. When the female *C. floridanum*-parasitized host hemolymph was added to the medium, all of the *G. pallipes* larvae died within 7 days, similar to male *C. floridanum*-parasitized host hemolymph (female *C. floridanum* alone: orange [mark]; together with *G. pallipes*: green; Fig. 4, Supplementary Fig. 1), indicating the same toxicity level as shown for males.

**Figure 5:**
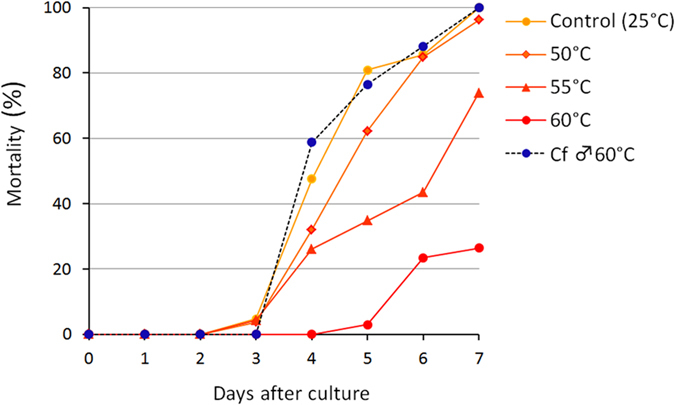
The thermal stability of the toxic activity of the experimental host hemolymph. Seven-day-old *G. pallipes* larvae were individually cultured in 20-μl drops of MGM450 medium. The mortality of *G. pallipes* larvae is shown for the female *C. floridanum*-parasitized host hemolymph heated at 50 °C (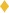), 55 °C (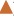) and 60 °C (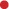) for 20 min and added to the medium to achieve a final concentration of 10%, together with the data for the non-heated control (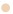) and the male *C. floridanum*-parasitized host hemolymph heated at 60 °C (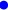, dashed line)^14^. For each sample, we used 35 to 40 *G. pallipes* larvae.

Hemolymph from female *C. floridanum*-parasitized hosts held toxicity even after heat treatment at 50 °C. However, the toxicity decreased as the temperature rose further, reaching only 26% at 60 °C (Fig. 5, Supplementary Fig. 2). This result markedly contrasts with the results of male *C. floridanum*–parasitized host hemolymph^14^, in which the toxicity was fully held, even at a 60 °C heat treatment (Fig. 5, dashed line). Importantly, the results of the proteinase K digestion (Supplementary Table 6) and the current heat treatment results suggest that the toxic factor(s) of female and male *C. floridanum* are different proteins.

**Figure 6:**
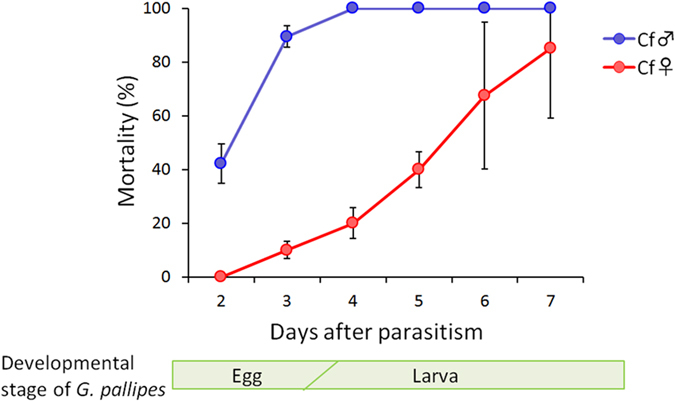
The mortality rate of *G. pallipes* in the growing hosts previously parasitized by male or female *C. floridanum*. The numbers of hosts used were shown in Supplementary Table 7.

When the larval parasitoid *G. pallipes* was allowed to parasitize the hosts previously parasitized by the egg-larval parasitoids of *C. floridanum*, the *G. pallipes* eggs and larvae might suffer damage from the soldier larvae and the toxic humoral factor of *C. floridanum*. Figure 6 shows the mortality rate of *G. pallipes* over the course of development. An obvious difference exists between the sexes of *C. floridanum*. When *G. pallipes* was multiparasitized with male *C. floridanum*, a high mortality rate of *G. pallipes* was observed during the egg stage (Fig. 6, Supplementary Table 7). In contrast, regarding female *C. floridanum*, the high mortality rate of *G. pallipes* appeared after hatching, which was most likely caused by soldier larvae.

## Discussion

**Figure 7:**
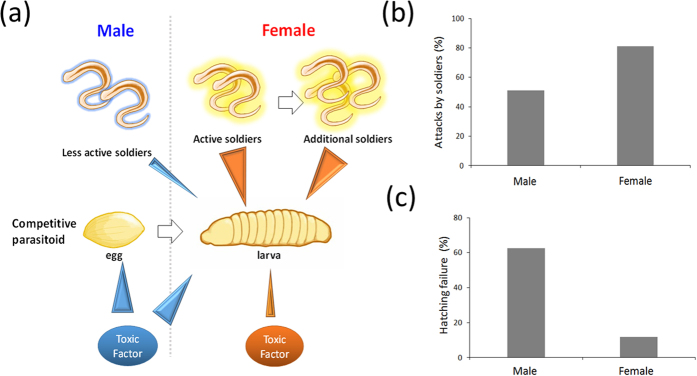
Sexual complementarity between male and female *C. floridanum*. **(a)** A schematic of the two defense systems (soldier larvae and toxic humoral factor). (**b**) Aggressiveness of male and female soldier larvae of *C. floridanum* against *G. pallipes* larvae *in vitro*^10^. (**c**) Hatching failure in *G. pallipes* in the host parasitized by male or female *C. floridanum* (data from Fig. 6).

The polyembryonic parasitoid *C. floridanum* has two defense traits against intrinsic heterospecific competitors: (1) soldier larvae^6^,^7^,^8^,^9^,^10^,^16^ and (2) toxic humoral factors^10^,^14^. This study provided the first evidence of the existence of humoral toxic factors produced by female *C. floridanum*, which were weaker than male factors. There are sex differences in the strength of toxic factors; male toxic factors are stronger than, but different from female ones. The current findings indicate that sexual complementarity exists between the two types of defense systems: (1) soldier larvae and (2) toxic factors of the sexes. The soldier larvae defense is strengthened in females, whereas the toxic humoral factor is strengthened in males (Fig. 7). Not only are female soldier larvae more aggressive than males^10^,^11^ but also the number of soldiers is increased upon invasion of the host by heterospecific competitors^17^,^18^. In contrast, the toxic factors produced by males not only kills the eggs of heterospecific competitors (that are not killed by female ones) but also exhibits high thermal stability against heat treatments. This sexual complementarity should strongly enhance the competitive ability of parasitoids in a mixed-sex brood where male and female eggs are laid on a host egg because of the much wider and stronger defense systems (Fig. 6). Such the strengthened defense capacity expected in a mixed-sex brood offers the possibility to explain the complementarity as an adaptive system.

The sexual complementarity that we found in the Japanese strain of *C. floridanum* should also provide an additional astonishing advantage of an apparent killing of male embryos by female soldier larvae for male wasps in a mixed-sex brood. When sexual differences in the wasp are discovered in the soldier larvae, sexual conflict is suspected because female soldiers kill many (perhaps a majority of) male reproductive (at embryo stages). This effect apparently drastically reduces male reproductive success and causes sexual conflict. If we consider the advantage of instant mating with all the emerging female reproductives from the same host, however, this view of sexual conflict is completely reversed. A male wasp (e.g., the behavior observed in fig wasps) should be able to inseminate hundreds of females^19^,^20^. Therefore, the few remaining male reproductives can immediately inseminate all of the female wasps that emerge from the same host. Note that a single host caterpillar should have the energetic and spatial capacity to produce emerging reproductives depending on their size^5^,^6^. By killing the male clone reproductive (embryos), the number of female reproductives can be increased drastically, approaching the total capacity of the reproductives. The number of female wasps inseminated by a wasp’s own clones can be increased by killing and removing most male embryos. Thus, the killing of male reproductives by female soldiers greatly enhances the reproductive success of both male and female wasps in a mixed brood. Strangely here, for the male embryos, ‘killed by female soldier larvae’ is highly adaptive, because their surviving clones can mate with many additional females that were never been grown if they survive. This adaptive advantage of instant mating also explains why mixed broods are highly common among wasps in the wild^10^,^18^,^21^.

Many reports have been made on the physiological suppression of parasitoids to eliminate competitors^22^,^23^,^24^,^25^. Our results suggest that the nature, composition, or both of the toxic factors differ between male and female *C. floridanum*. To our knowledge, the current finding is the first report of the sexual differences concerning the physiological suppression of competitors by parasitic wasps. Importantly, the male toxic factor cannot damage conspecific reproductives, but it increases the defense against heterospecific competitors, guaranteeing the advantage of mixed-sex broods.

The toxicity of the humoral factor appears to be due to paralytic action because *G. pallipes* larvae became motionless after injecting the host hemolymph into the host parasitized by *G. pallipes* alone, with recovery occurring later (Fig. 3). This observation suggests that the toxic factor is easily denatured or metabolized in the host hemolymph. Within the multiparasitized host, however, *G. pallipes* larvae must suffer incessant damage from the toxic factor continuously produced by *C. floridanum*; eventually, they die within the host. Moreover, the toxic factor might be relatively stable under culture conditions because *G. pallipes* larvae cultured in the medium supplemented with *C. floridanum*-parasitized host hemolymph died without recovering movement (Fig. 4).

Our recent study showed that male *C. floridanum* cancels the host-immune suppression of *G. pallipes*, whereas their female counterparts do not^26^. The humoral factor produced by the male *C. floridanum*–parasitized hosts might be multifunctional or multicomponent and related to host immune enhancements. Nevertheless, the mechanism of the ovicidal activity regarding the toxic factor in male *C. floridanum*–parasitized hosts remains unknown. Furthermore, to date, the question whether the toxic factor is directly produced by *C. floridanum* embryos or indirectly from host tissues remains unresolved. In the former case, male and female *C. floridanum* embryos may produce male- and female-specific toxic factors, respectively. In contrast, in the later case, *C. floridanum* embryos release male- and female-specific molecules which induce the production of different toxic factors according to the sexes of *C. floridanum* by host tissues. Future biochemical study may answer these questions.

Striking differences exist between the Japanese and American strains of *C. floridanum*. In the latter strain, male soldiers are functionless and appear only in the final host instar^7^,^8^,^27^. A male single brood of the American strain fails to emerge when heterospecific multiparasite competition exists^11^. This finding indicates the lack of an effective toxic humoral factor in this strain (unlike the Japanese strain). Two plausible interpretations exist for the loss of the competitive ability in American strain males. First, few or no heterospecific competitors might exist in the USA. However, this interpretation seems unlikely because multiparasitism should be fairly common^5^.

The second interpretation is more likely: Male wasps from a single male brood have almost no chance of mating with female wasps because of the difficulty of locating a mate. Unlike the small mosaic habitats of the Japanese strain, American strain wasps are found in vast agricultural farmlands. There, the chance of outbreeding is mostly negligible for both male and female single broods. Thus, the reproductive success of the American strain is almost exclusively guaranteed by inbreeding within the mixed-sex broods that are at least competitively superior to heterospecific competitors. This supposition also implies that the American strain loses the competitive ability associated with a single host when they first disperse in their area of origin, where the reproduction of single broods is at least guaranteed (as in the Japanese strain). This hypothesis also accounts for why the Japanese strain has a competitive ability in both males and females (Fig. 7). If this interpretation is true, then the American strain should be more genetically homozygous than the Japanese strain.

## Methods

### Materials

*Acanthoplusia agnata* (Lepidoptera: Noctuidae) was used as the host for both *Copidosoma floridanum* (Hymenoptera: Encyrtidae) and *G. pallipes* (Hymenoptera: Braconidae). *A. agnata* larvae were collected in Tokyo Prefecture and then successively raised on an artificial diet at 25 °C (L:D 16:8 h). Adult insects were fed a 10% sugar solution absorbed in cotton. *C. floridanum* adults were obtained from parasitized Plusiinae larvae (*A. agnata*, *C. eriosoma* and *Thysanoplusia intermixta*) from burdock fields in Tokyo Prefecture and Chiba Prefecture. The eggs laid by *A. agnata* (within 24 h post-oviposition) were used for parasitism by *C. floridanum*. The parasitized hosts were kept in the same conditions as the non-parasitized hosts. One-day-old third-instar larvae (L3D1) of *A. agnata* were used for *G. pallipes* parasitism. Multiparasitism was accomplished as follows: 24-h-old eggs of *A. agnata* were parasitized by *C. floridanum* and subsequently parasitized by *G. pallipes* at the host’s L3D1 larval stage.

### Collection of hemolymph

Host hemolymph was collected during the host’s L6D1 stage from pierced abdominal prolegs of non-parasitized and parasitized *A. agnata* larvae that had been surface-sterilized with 70% ethanol solution. To prevent the melanization of the hemolymph, phenylthiourea was added to achieve a final concentration of 0.01% immediately after the hemolymph was drawn. Hemocytes were then removed via centrifugation at 1,000 × *g* for 20 min at 4 °C, and the supernatant was stored at −40 °C until use.

### *In vivo* assay with *G. pallipes* eggs

To examine the *in vivo* toxicity of host hemolymph to *G. pallipes* eggs, we injected 6 μl of *C. floridanum*-parasitized or non-parasitized host hemolymph into the L3D1 host larvae immediately after parasitism by *G. pallipes*. *G. pallipes* is a multiparasitoid that lays approximately 80 eggs per host in a single oviposition bout. After 84 h of parasitism, we dissected the host larvae, counted the number of hatched and unhatched *G. pallipes* eggs, and calculated the hatchability. The effect of the injection of host hemolymph during the egg stage of *G. pallipes* upon successful parasitism was also investigated. In this experiment, the hemolymph-injected and non-injected hosts were maintained until host pupation (failure of parasitism) or *G. pallipes* larval egression (successful parasitism).

### *In vivo* assay with *G. pallipes* larvae

*In vivo* assay was performed via the injection of hemolymph into the hosts with *G. pallipes* larvae. *G. pallipes* females were allowed to parasitize L3D1 host larvae. To examine the toxicity of the host hemolymph to *G. pallipes* larvae, we injected 6 μl of *C. floridanum*-parasitized or non-parasitized host hemolymph into the host larvae 72 h post-parasitism. The hemolymph-injected host larvae were dissected, and the numbers of moving and non-moving *G. pallipes* larvae were counted daily for four days.

### *In vitro* assay

*In vitro* assays were performed via the hemolymph treatment of *G. pallipes* larvae in culture. The *G. pallipes* larvae were removed from the host larvae and placed in the culture medium (MGM450 medium) seven days after parasitism and during the host’s L3D1 stage. After being washed with a fresh culture medium four times, these larvae were transferred into a 20 μl drop of the medium supplemented with or without 10% parasitized host or non-parasitized host hemolymph and placed in a plastic 35-mm Petri dish. The cultures were maintained at 25 °C, and larval mortality was examined daily for seven days. The assays were performed in conjunction with our previous experiments^14^.

## Additional Information

**How to cite this article**: Uka, D. *et al*. Sexual complementarity between host humoral toxicity and soldier caste in a polyembryonic wasp. *Sci. Rep*. **6**, 29336; doi: 10.1038/srep29336 (2016).

## Supplementary Material

Supplementary Information
