# Supplementary material for: Sexual complementarity between host humoral toxicity and soldier caste in a polyembryonic wasp
Source: Sci Rep. 2016 Jul 7;6:29336. doi: 10.1038/srep29336 (PMC4935867; doi:10.1038/srep29336)
Supplement: Supplementary Information [file srep29336-s1.pdf]

Supplementary Information for

Sexual complementarity between host humoral toxicity and soldier caste in a polyembryonic wasp

Daisuke Uka, Takuma Sakamoto, Jin Yoshimura and Kikuo Iwabuchi

This PDF file includes:

Supplementary Figures 1 and 2

Supplementary Tables 1-7

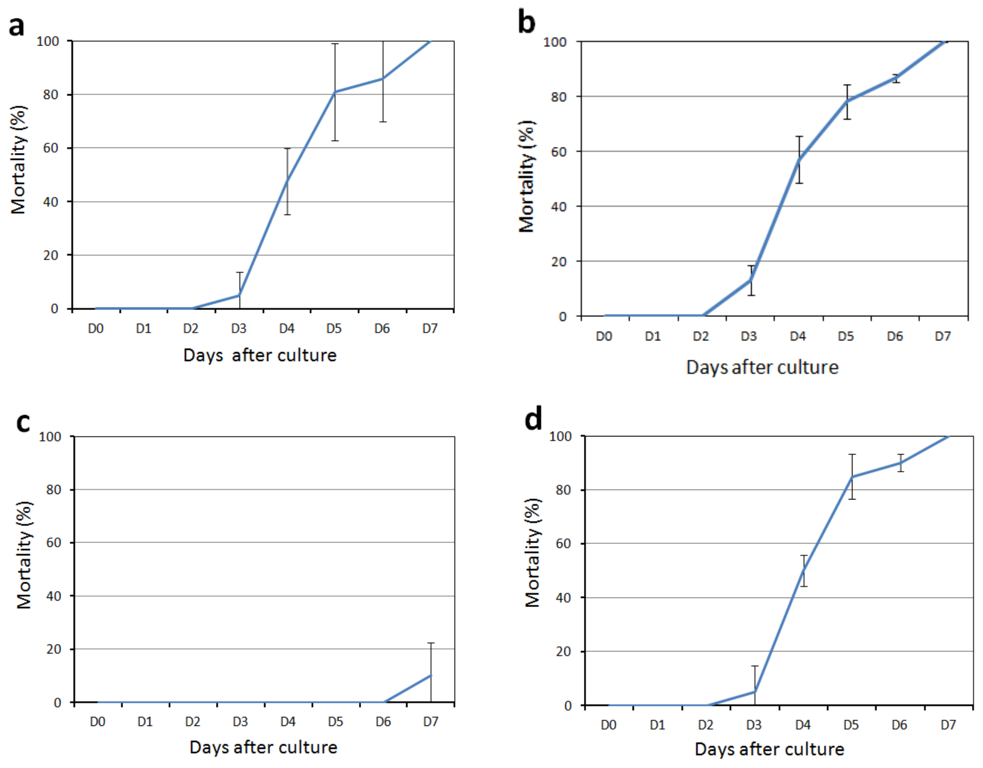

Supplementary Fig. 1. Percentage mortality figures for each treatment, represented with SD bars. **a**, Cf♀-parasitized; **b**, Cf♀+ *G. pallipes*- multiparasitized; **c**, non-parasitized; **d**, Cf♂-parasitized host hemolymph (related to Fig. 2a).

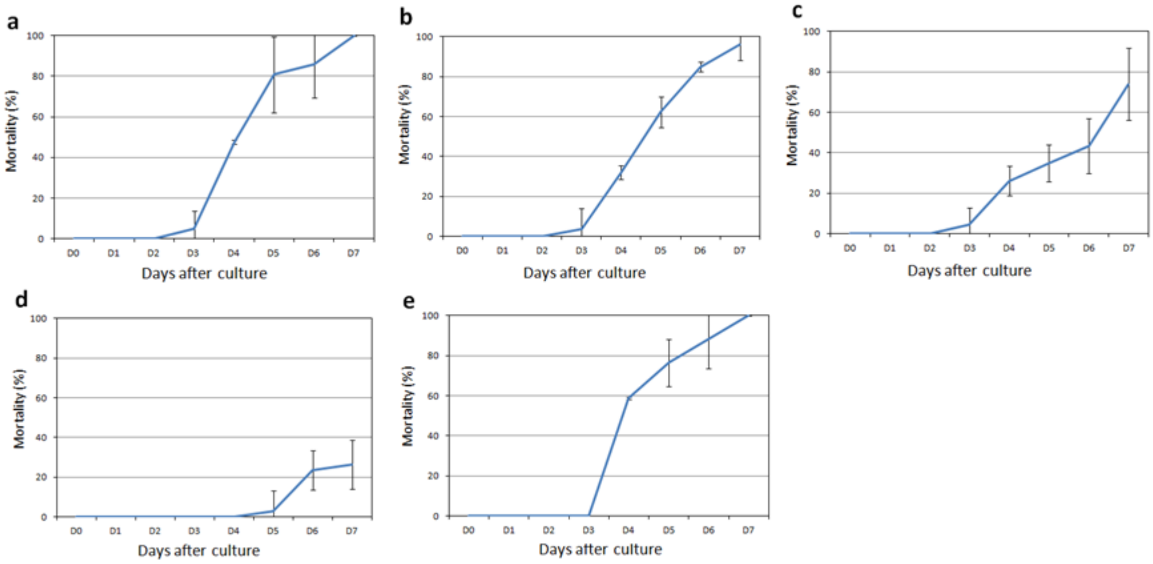

Supplementary Fig. 2. Percentage mortality figures for each treatment, represented with SD bars. **a**, control (25°C); **b**, 50°C; **c**, 55°C; **d**, 60°C; **e**, Cf♂(60°C) (related to Fig. 2**b**).

Supplementary Table 1. State of the hosts for hemolymph samples and the effect of hemolymph injection into *G. pallipes*-parasitized hosts on hatchability of the eggs.(related to Fig. 1a)

1 non-parasitized hosts

Host #	No. of Gp eggs			Hatchability(%)
	Hatched	Unhatched	Total	
1	75	12	87	86.2
2	84	5	89	94.4
3	59	0	59	100.0
4	118	12	130	90.8
5	84	6	90	93.3
6	49	8	57	86.0
7	67	4	71	94.4
8	55	5	60	91.7
9	81	7	88	92.0
10	74	6	80	92.5
11	60	4	64	93.8
12	67	5	72	93.1
13	67	3	70	95.7
14	80	9	89	89.9
15	99	12	111	89.2
16	127	14	141	90.1
17	73	10	83	88.0
18	66	10	76	86.8
19	95	9	104	91.3
20	93	15	108	86.1
21	71	9	80	88.8
22	64	11	75	85.3
23	88	19	107	82.2
24	84	7	91	92.3
25	79	9	88	89.8
26	78	17	95	82.1
27	96	20	116	82.8
28	119	11	130	91.5
29	100	8	108	92.6
30	87	16	103	84.5

2 Cf ♂ -parasitized

Host #	No. of Gp eggs			Hatchability(%)
	Hatched	Unhatched	Total	
1	15	66	81	18.5
2	86	21	107	80.4
3	8	84	92	8.7
4	42	5	47	89.4
5	9	63	72	12.5
6	6	67	73	8.2
7	8	78	86	9.3
8	25	87	112	22.3
9	28	41	69	40.6
10	61	15	76	80.3
11	12	78	90	13.3
12	7	58	65	10.8
13	20	98	118	16.9
14	8	50	58	13.8
15	11	100	111	9.9
16	72	17	89	80.9
17	79	12	91	86.8
18	26	68	94	27.7
19	27	77	104	26.0
20	66	5	71	93.0

3 Cf ♀ -parasitized

Host #	No. of Gp eggs			Hatchability(%)
	Hatched	Unhatched	Total	
1	91	1	92	98.9
2	84	17	101	83.2
3	68	7	75	90.7
4	57	4	61	93.4
5	51	8	59	86.4
6	33	13	46	71.7
7	74	19	93	79.6
8	112	14	126	88.9
9	110	7	117	94.0
10	45	1	46	97.8
11	45	14	59	76.3
12	42	0	42	100.0
13	92	4	96	95.8
14	106	3	109	97.2
15	68	1	69	98.6
16	111	12	123	90.2
17	53	14	67	79.1
18	78	12	90	86.7
19	51	3	54	94.4
20	37	27	64	57.8
21	48	9	57	84.2
22	44	14	58	75.9
23	38	1	39	97.4
24	92	4	96	95.8
25	81	8	89	91.0

4 Gp -parasitized

Host #	No. of Gp eggs			Hatchability(%)
	Hatched	Unhatched	Total	
1	99	1	100	99.0
2	64	12	76	84.2
3	88	13	101	87.1
4	56	4	60	93.3
5	76	8	84	90.5
6	94	13	107	87.9
7	104	19	123	84.6
8	52	4	56	92.9
9	37	7	44	84.1
10	102	1	103	99.0
11	81	9	90	90.0
12	49	0	49	100.0
13	52	5	57	91.2
14	53	3	56	94.6
15	34	1	35	97.1
16	53	12	65	81.5
17	103	14	117	88.0
18	105	12	117	89.7
19	40	3	43	93.0

Supplementary Table 2. Statistical tests using Welch's two sample t-test for egg mortality of *G. pallipes* in the host injected with test host hemolymph (related to Fig. 1a)

Between two test hemolymph	t-value	df	p-value
Non-parasitized / Cf ♂-parasitized	7.1839	48	5.36e-07
Cf ♂-parasitized/ Cf ♀-parasitized	6.744	43	4.63e-07
Non-parasitized / Cf ♀-parasitized	0.1280	53	0.8989
Non-parasitized / Gp-parasitized	-7.259	47	2.17e-07

Supplementary Table 3. Contingration test - Observed (and expected) frequencies of successful and failed parasitism in *G. pallipes* in the hosts injected with test host hemolymph at parasitism. (related to Fig. 1b)

Host hemolymph compared	Successful parasitism	Failed parasitism	Totals
Non-parasitized	22 (20.7)	8 (9.3)	30
Cf ♀	16 (17.3)	9 (7.7)	25
Totals	38	17	55

X2 = 13.675, df=1, p<0.001

Host hemolymph compared	Successful parasitism	Failed parasitism	Totals
Cf ♂	4 (8.9)	16 (11.1)	20
Cf ♀	16 (10.4)	9 (9.6)	25
Totals	20	25	45

X2 = 8.712, df=1, p<0.01

Host hemolymph compared	Successful parasitism	Failed parasitism	Totals
Non-parasitized	22 (15.6)	8 (14.4)	30
Cf ♂	4 (10.4)	16 (9.6)	20
Totals	26	24	50

X2 = 0.5562, df=1, p>0.05

Supplementary Table 4. Number of samples (hosts) used in the experiments shown in Fig. 1c.

Hemplymph of hosts	Time after hemolymph injection 24h	48h	72h	96h
Non-parasitized	20	20	20	20
Parasitized by				
Cf♂	20	20	20	20
Cf♀	25	25	25	25
Cf♀ + Gp	25	25	25	25

Supplementary Table 5. Statistical tests using Welch's two sample t-test for larval moving activity of *G. pallipes* in the host injected with test host hemolymph (related to Fig. 1c)

Between two test hemolymph (time after injection)	t-value	df	p-value
Cf♂ (24h)/ Cf♂ (48h)	-0.6506	32.470	0.5199
Cf♂ (48h)/ Cf♂ (72h)	-12.331	25.936	2.371e-12
Cf♂ (72h)/ Cf♂ (96h)	-4.3621	28.103	0.0002
Cf♂ (24h)/ Cf♂ (96h)	-22.378	35.216	<2.2e-16
Cf♂ (48h)/ Cf♂ (96h)	-27.507	37.177	<2.2e-16
Non(24h)/ Cf♂ (24h)	23.079	29.627	<2.2e-16
Cf♂ (24h)/ Cf♀ (24h)	-20.154	36.541	<2.2e-16
Cf♂ (24h)/ Cf♀ +Gp(24h)	-22.789	33.052	<2.2e-16

Supplementary Table 6. Effect of proteinase on the toxicity of the hemolymph

Proteinase	Concentration (μg/ml)	No. of hosts	Mortality (%)
Proteinase K	1.25	30	33.3
	2.5	28	14.3
	5	25	8
	10	31	4.2

Hemolymph collected from *C. floridanum* -parasitized hosts was pretreated at 60°C from 20 min.
One ml of Proteinase K solution was added to 1oo ml of hemolymph at 25°C for 60 min.
The reaction was terminated by the addition of PMSF.
Mortality of *G. pallipes* was calculated 7 days after incubation.

Supplementary Table 7. Number of samples examined for changes in mortality of *G. pallipes* multiparasitized with male or female *C. floridanum* during development (related to Fig. 3b)

Multiparasitized with	Days after Gp parasitism					
	D2	D3	D4	D5	D6	D7
Cf♂	45	38	38	35	37	37
Cf♀	35	40	35	35	40	40
